# Preferential Activation of SMAD1/5/8 on the Fibrosa Endothelium in Calcified Human Aortic Valves - Association with Low BMP Antagonists and SMAD6

**DOI:** 10.1371/journal.pone.0020969

**Published:** 2011-06-15

**Authors:** Randall F. Ankeny, Vinod H. Thourani, Daiana Weiss, J. David Vega, W. Robert Taylor, Robert M. Nerem, Hanjoong Jo

**Affiliations:** 1 Wallace H. Coulter Department of Biomedical Engineering, Georgia Institute of Technology and Emory University, Atlanta, Georgia, United States of America; 2 Division of Cardiothoracic Surgery, Emory University, Atlanta, Georgia, United States of America; 3 Division of Cardiology, Emory University, Atlanta, Georgia, United States of America; 4 Petit Institute for Bioengineering and Bioscience, Georgia Institute of Technology, Atlanta, Georgia, United States of America; 5 Department of Bioinspired Science, Ewha Woman's University, Seoul, Korea; Fundació Institut Germans Trias i Pujol; Universitat Autònoma de Barcelona CibeRES, Spain

## Abstract

**Background:**

Aortic valve (AV) calcification preferentially occurs on the fibrosa side while the ventricularis side remains relatively unaffected. Here, we tested the hypothesis that side-dependent activation of bone morphogenic protein (BMP) pathway in the endothelium of the ventricularis and fibrosa is associated with human AV calcification.

**Methods and Results:**

Human calcified AVs obtained from AV replacement surgeries and non-calcified AVs from heart transplantations were used for immunohistochemical studies. We found SMAD-1/5/8 phosphorylation (a canonical BMP pathway) was higher in the calcified fibrosa than the non-calcified fibrosa while SMAD-2/3 phosphorylation (a canonical TGFβ pathway) did not show any difference. Interestingly, we found that BMP-2/4/6 expression was significantly higher on the ventricularis endothelium compared to the fibrosa in both calcified and non-calcified AV cusps; however, BMP antagonists (crossvienless-2/BMPER and noggin) expression was significantly higher on the ventricularis endothelium compared to the fibrosa in both disease states. Moreover, significant expression of inhibitory SMAD-6 expression was found only in the non-calcified ventricularis endothelium.

**Conclusions:**

SMAD-1/5/8 is preferentially activated in the calcified fibrosa endothelium of human AVs and it correlates with low expression of BMP antagonists and inhibitory SMAD6. These results suggest a dominant role of BMP antagonists in the side-dependent calcification of human AVs.

## Introduction

Aortic valve (AV) disease is a major cause of cardiac deaths worldwide and is a strong risk factor for additional cardiovascular events [1], [2], [3]. With the aging United States population, it is believed that 20% of individuals over the age of 80 have AV calcification, making it the most common cardiac disease [4]. AV calcification was once thought to be a passive degenerative disease but is now known as an active inflammatory pathology [5], [6], [7]. AV calcification is characterized by the accumulation of calcium, inorganic phosphates, extracellular matrix proteins, bone-related factors [8], [9], [10], and osteoblast-like cells [8], [11] in the fibrosa, or aortic side, of the valve cusp [10], [12].

The AV is comprised of three distinct layers: the fibrosa, ventricularis, and spongiosia. The fibrosa, which faces the aorta, is comprised of collagen fibers, while the ventricularis faces the left ventricle and is comprised of elastin and collagen fibers. Finally, the spongiosia, which is located in between the fibrosa and ventricularis, is comprised of glycosaminoglycans [13]. A continuous endothelial monolayer covers the valve, while a healthy valvular leaflet contains a heterogeneous population of valvular interstitial cells [13]. The AV resides in a complex mechanical environment that includes fluid shear stresses, varying pressures, and bending stresses [14]. Similar to the vascular endothelial system, where atherosclerosis preferentially occurs in areas of disturbed flow, AV calcification and sclerosis primarily occur in a side-dependent manner [15], [16], [17], [18], [19]. The fibrosa endothelium experiences disturbed flow conditions throughout the cardiac cycle and is prone to accelerated AV calcification. Conversely, the ventricularis endothelium experiences stable flow during systole and remains relatively unaffected. The correlation between hemodynamic forces and AV disease development suggests that the AV endothelium may be playing a role in AV disease development. Recent studies performed by our group and others have begun to investigate the endothelium's role in AV valve biology. In a study looking at side-specific mRNA of the AV endothelium of porcine AVs, Simmons et al. found the pro-inflammatory and bone growth chemokine bone morphogenic protein 4 (BMP-4) was expressed on the fibrosa endothelium, while chordin, a natural BMP antagonist, was found to be up-regulated on the ventricularis endothelium. This suggests a pro- and anti-osteogenic conditions on the fibrosa and ventricularis sides respectively [20]. Butcher, et al. found that porcine AV endothelial cells, when exposed to unidirectional laminar flow, decreased BMP-4 expression [15]. Furthermore, BMP-4 expression was higher in the fibrosa of porcine AV compared to the ventricularis (14). It was also reported that BMPs -2 and -4 are present in calcified regions of human AV [21]. However, it is not known whether BMPs are activated in endothelial cells in a side-dependent manner and whether it correlates with calcification in human AVs.

BMPs are members of the TGFβ superfamily. Initially discovered as inducers of bone formation [8], the BMPs are now known to play important roles in embryonic development, patterning, cartilage formation, and cell differentiation [22], [23]. We have shown that BMP-4 is a mechanosensitive and proinflammatory cytokine in vascular endothelial cells [24], [25]. Furthermore, BMP-4 infusion induced hypertension in mice in a NADPH oxidase-dependent manner [26]. In addition, BMP-2 and -4 expression is increased in calcified human AVs and human atherosclerotic lesions [21], [27].

Another classification of molecules, BMP antagonists, bind to the BMPs with varying degrees of affinity. Once bound, BMP antagonists inhibit the interaction of the BMPs with their cognate receptors [28], [29], [30], [31], [32], [33]. BMP antagonists include, noggin, crossveinless 2 (CV-2, also known as BMPER), chordin, follistatin, DAN and matrix Gla protein-1(MGP-1) [34]. In porcine AV leaflets, chordin was increased on the ventricularis endothelium [20]. Interestingly, uncarboxylated MGP-1 is decreased in the plasma of human patients that have AV calcification versus the healthy cohort [35].

The BMPs and TGFβ have two types of specific signaling receptors: BMPR-I and BMPR-II, or TGFβR-1 and TGFβR-II, respectively, and both are required for signaling. Once the ligand is bound to its receptors, the active domain of the type II receptor phosphorylates the type I receptor, which in turn phosphorylates the R-SMADs (phospho-SMAD 1, 2, 3, 5, 8) [36], [37]. SMAD-2/3 and SMAD-1/5/8 are canonical mediators of TGFβ and BMP signaling, respectively. These phospho-SMADs then bind with co-SMAD-4 and translocate into the nucleus, regulating a wide range of gene expression. SMAD-6, an inhibitory SMAD, can block the R-SMADs from being phosphorylated by competing for activation of the type I receptors [36], [37].

At present, it is not known whether BMPs and BMP antagonists play a role in human AV calcification. We hypothesized that BMP pathway is preferentially activated in the fibrosa endothelium, which leads to side-specific human AV calcification in the fibrosa. Our current study using calcified and non-calcified human AVs show that phospho-SMAD-1/5/8 is significantly higher in the fibrosa endothelium, suggesting that BMP pathway is preferentially activated in the fibrosa endothelium of calcified human AVs. Our studies further show that the fibrosa-dependent activation of the BMP pathway correlates well with decreased levels of BMP antagonists and inhibitory SMAD-6.

## Materials and Methods

### Human AV Procurement

AVs were received from two patient populations according to the IRB-approved study at Emory University with written informed consent. Calcified human AVs were obtained immediately following valve replacement surgeries in 16 patients at Emory University Hospital Midtown. Fifteen patients had trileaflet valves, while one patient had a bicuspid AV. Non-calcified AV (n = 6, all trileaflet AV) were harvested from recipient patients undergoing heart transplantation at Emory University Hospital. Two of the six patients underwent cardiac transplantation due to ischemic cardiomyopathy. Patient demographics are presented in Table 1. Immediately following harvesting, the AVs were photographed, washed in ice-cold phosphate buffered saline, and cusps were individually snap-frozen in optimal cutting temperature (O.C.T.) compound (Tissue-Tek). Valves were then sectioned (7 µm) in the radial direction to include the base and free edge (tip), stored at −80°C and used for immunohistochemical staining studies.

**Table 1:** Patient Characteristics.

	Calcified	Non-Calcified
Number of Patients	16	6
Age (mean ± SD)	66.4±16.28	54.7±8.5
Female	7 (43.8%)	1 (16%)
Bicuspid valves	1 (6.25%)	0 (0%)
Ejection Fraction (mean ± SD)	0.498±0.139	NA
NYHA* (mean ± SD)	2.2±1.1	NA
Congestive Heart Failure	9 (56.25%)	6 (100%)
Diabetes mellitus	2 (12.5%)	3 (50%)
Hemoglobin A1C (mean ± SD)	6±1.18	NA
Dyslipidemia	6 (37.5%)	NA
Hypertension	12 (75%)	2 (33.3%)
Last Creatinine Level (mean ± SD)	1.12±0.33	NA
Dialysis	0 (0%)	NA

*NYHA - New York Heart Association.

### Histochemistry and Immunohistochemistry

Hematoxalin and eosin (H&E for general histology), Verhoeff Van Giessen (for elastin), and Alizarin Red (for calcification) staining was carried out for histomorphometric analysis. Immunohistochemical studies were carried out as previously described [34] using the following specific antibodies: endothelial marker; von Willebrand Factor or vWF, (Dako, 1∶400), BMPs; BMP-2 (Lifespan Biosciences, 1∶100), BMP-4 (Biovision, 1∶25), and BMP-6 (Santa Cruz. 1∶25), BMP antagonists; noggin (LabFrontier, 1∶100), CV-2/BMPER (R&D, 1∶100), MGP-1 (ABCAM, 1∶100) and DAN (R&D, 1∶25), phospho-SMAD-1/5/8 (Cell Signaling, 1∶200) and phospho-SMAD-2 (Cell Signaling, 1∶100), and SMAD-6 (Lifespan Biosciences 1∶25). Rhodamine Red X antibody (Jackson Labs) was used as a secondary antibody with a Hoechst dye nuclear counter staining. Fluorescent images were taken with a Zeiss Axioskop epifluorescence microscope using a 10× objective.

### Image Analysis

Three cross-sectional images were obtained from each AV section, where endothelial layer staining was present based on Hoechst staining. Digital images were then graded for endothelial staining intensity from 0 (no positive staining) to 5 (most intense positive staining) by three blinded individuals. The grades of the three cross-sections were averaged to determine the staining intensity of each antibody examined. The fibrosa and ventricularis endothelium were graded separately.

### Statistical Analysis

All data are reported by mean ± SE with n signifying the number of different AV leaflets stained. Significant differences were determined by ANOVA using a Tukey HSD testing. All p-values<0.05 were considered significant.

## Results

### Immunohistochemical examination of AVs

**Figure 1:**
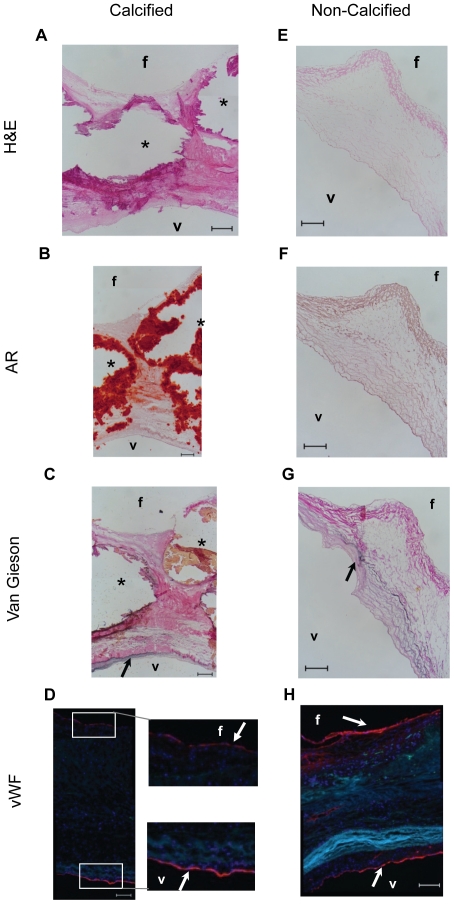
Calcification and endothelial staining of AV cusps. Valves were obtained from either heart transplant (non-calcified) or valve replacement (calcified) surgeries, snap frozen and sectioned. Sections were then stained for H&E (**A**, **E**), alizarin red (**B**, **F**), Verhoeff Van Giessen (**C**, **G**) or von Willebrand factor (**D**, **H**). Representative staining (n = 12 patients) shows side-specific calcification (*) in calcified leaflets (**B**, **F**), while maintaining an intact endothelial layer (**D**, **H**: arrows). Verhoeff Van Giessen stain was used to stain for elastin (shown in black, arrows) to help in leaflet orientation (**C**, **G**). f: fibrosa, v: ventricularis.

H&E staining for general histology (Figure 1A,E), Alizarin Red (Figure 1B,F) for calcification and Verhoeff Van Giessen (Figure 1C,G) for elastin was carried out with calcified and non-calcified human AVs (Table 1). All human AVs (n = 6 patients) obtained from the heart transplantation patients were negative for Alizarin Red staining (Fig. 1F), suggesting that they were not calcified. In contrast, all calcified AVs (n = 16 patients) obtained from AV replacement surgeries were confirmed by Alizarin Red staining (Figure 1B). To examine the presence of an intact endothelium, von Willebrand factor staining was performed on the AV leaflet (Figure 1D, H). Intact endothelium was confirmed on valves used in our study.

### Preferential activation of BMP-dependent SMAD1/58 pathway in calcified human AV

**Figure 2:**
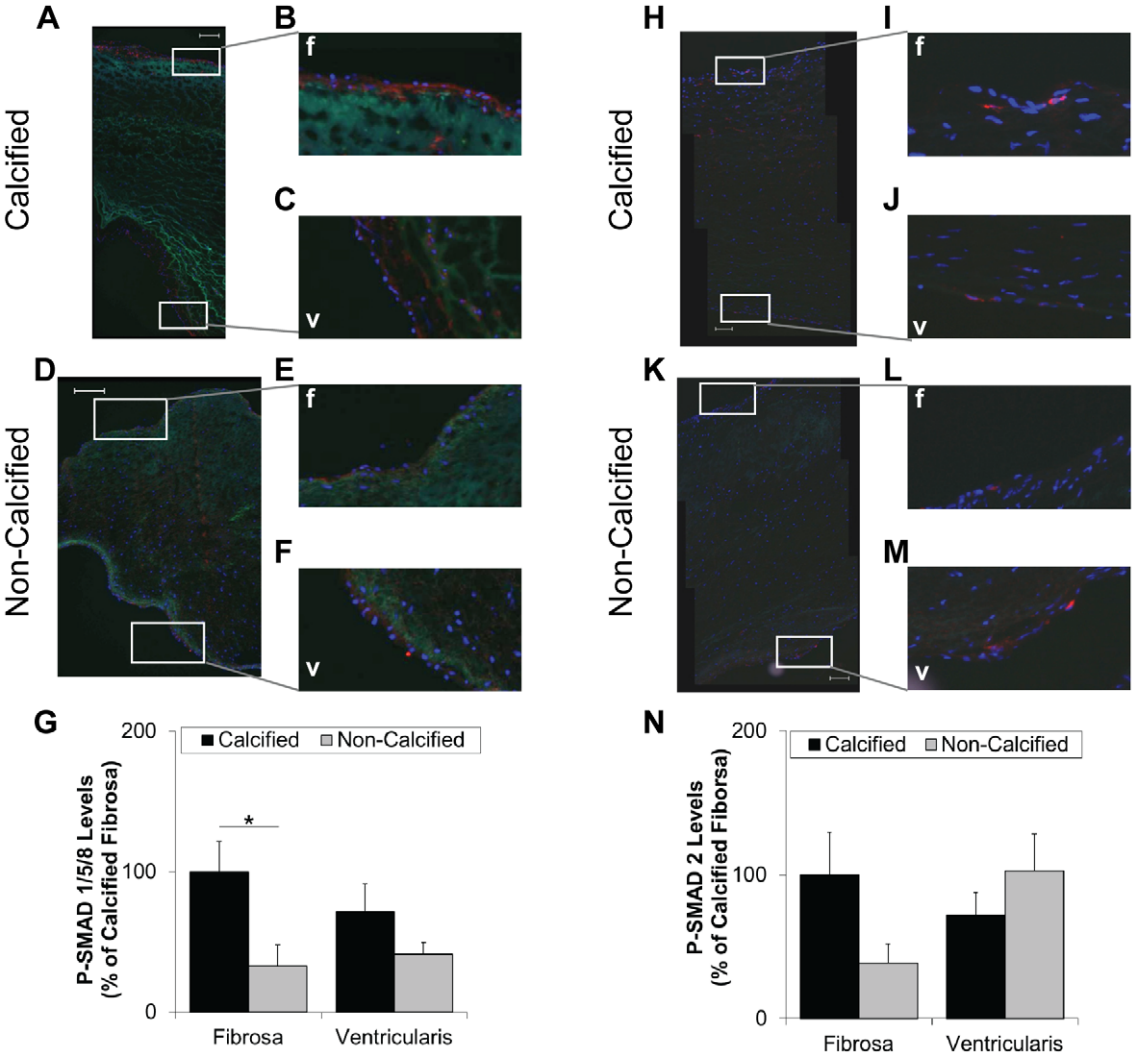
Phospho-SMAD 1/5/8 level is high in calcified fibrosa endothelium. Calcified and non-calcified AV sections were stained for phospho-SMAD 1/5/8 (**A–F**), and phospho-SMAD 2 (**H–M**), and a rhodamine-labeled secondary antibody. Shown are representative images. Bar graphs show staining intensities of fibrosa- and ventricularis-endothelium for each SMAD (**G**, **N**) (mean+SEM). For phospho-SMAD 1/5/8, n = 13 calcified and n = 12 non-calcified. For phospho-SMAD 2, n = 14 calcified and n = 13 non-calcified. For SMAD-6, n = 22 calcified and n = 15 non-calcified. *p<0.05, #p<0.06. f: fibrosa, v: ventricularis.

To determine whether the BMP pathway is activated in human AV endothelium in a side- dependent manner, we examined the level of phospho-SMAD-1/5/8, a canonical BMP activation pathway marker. Intense phospho-SMAD-1/5/8 staining was observed only in the calcified fibrosa endothelium (Fig. 2A–G). In contrast, non-calcified AV endothelium in both fibrosa and ventricularis showed only faint levels of phospho-SMAD-1/5/8. While the phospho-SMAD-1/5/8 staining level was low in the calcified ventricularis, it did not reach statistical significance in comparison to the calcified fibrosa (Fig. 2G).

As a comparison, phospho-SMAD-2 levels, a canonical TGFβ signaling activation marker, was studied. Overall, we did not observe any statistically significant differences in phospho-SMAD-2 levels in any of the AV endothelial groups; however, we found a trend for lower phospho-SMAD-2 levels in the non-calcified fibrosa endothelium (p<0.1, n = 13) compared to the non-calcified ventricularis endothelium (Fig. 2 H–N).

### Side-dependent expression of BMPs in human AV

**Figure 3:**
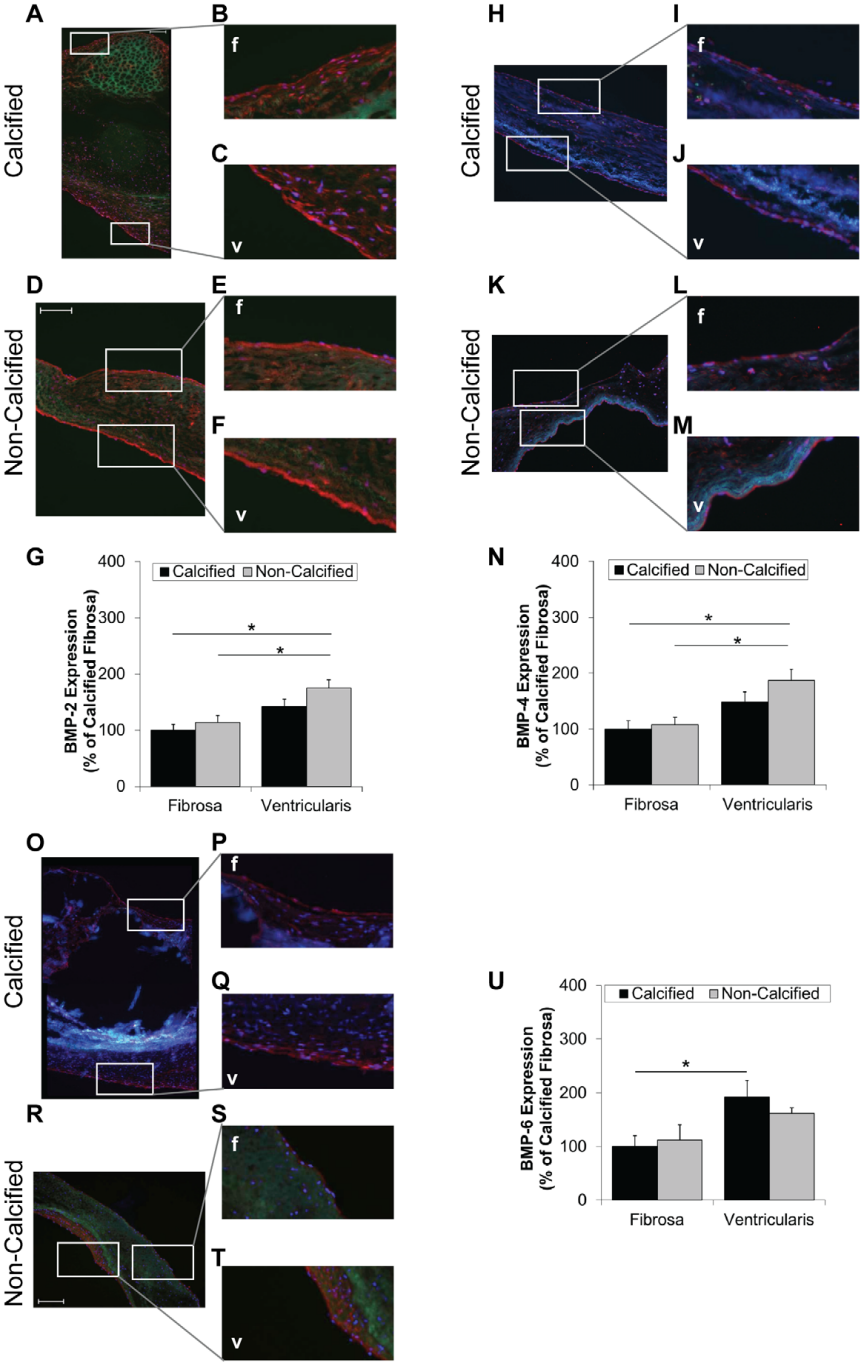
BMP expression in the fibrosa and ventricularis endothelium. Calcified and non-calcified AV sections were stained for BMP-2 (**A–F**), BMP-4 (**H–M**), and BMP-6 (**O–T**), and a rhodamine-labeled secondary antibody. Shown are representative images. Bar graphs show staining intensities of fibrosa- and ventricularis-endothelium for each BMP (**G**, **N**, **U**) (mean+SEM). For BMP-2, n = 13 calcified and n = 13 non-calcified. For BMP-4, n = 9 calcified and n = 8 non-calcified. For BMP-6, n = 12 calcified and n = 11 non-calcified. *p<0.05. f: fibrosa, v: ventricularis.

Next, we determined whether the side- and calcification-dependent phosphorylation of SMAD-1/5/8 correlates with BMP expression levels. Robust expression of BMPs -2, -4, and -6 was observed in all the tested AV endothelium. To our surprise, based on previous studies using healthy porcine AVs [15], [20], BMP-2, -4, and -6 expression was higher in ventricularis endothelium than fibrosa endothelium (Figure 3). BMP-2 and -4 expression was significantly higher in non-calcified ventricularis endothelium compared to the fibrosa endothelium of both calcified and non-calcified AVs (Figure 3 A–N). BMP-6 expression was significantly higher in the calcified ventricularis endothelium than the fibrosa endothelium (Figure 3 O–U). There was no significant difference in expression levels of all three BMPs in the ventricularis endothelium of calcified and non-calcified AVs. The same was true for the fibrosa endothelium of calcified and non-calcified AVs (Figure 3).

### Side-dependent expression of BMP antagonists and the inhibitory SMAD-6 in human AV

**Figure 4:**
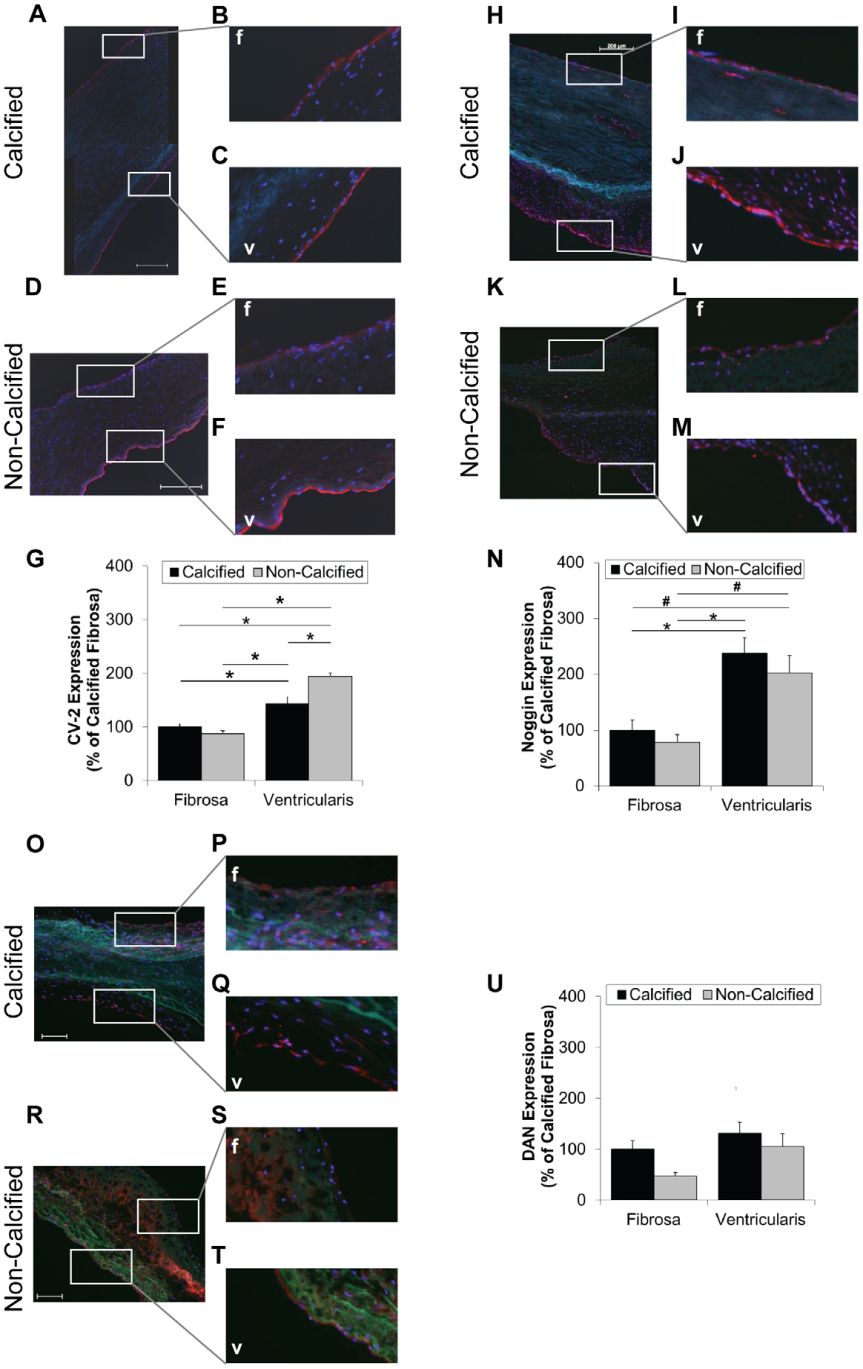
BMP antagonist expression in the fibrosa and ventricularis endothelium. Calcified and non-calcified AV sections were stained for CV-2/BMPER (**A–F**), noggin (**H–M**), DAN (**O–T**), and SMAD-6 (**V–BB**), and a rhodamine-labeled secondary antibody. Shown are representative images. Bar graphs show staining intensities of fibrosa- and ventricularis-endothelium for each antagonist (**G**, **N**, **U**, **BB**) (mean+SEM). For CV-2/BMPER, n = 20 calcified and n = 14 non-calcified. For noggin, n = 14 calcified and n = 6 non-calcified. For DAN, n = 10 calcified and n = 8 non-calcified. For SMAD-6, n = 22 calcified and n = 15 non-calcified. *p<0.05, f: fibrosa, v: ventricularis.

**Figure 5:**
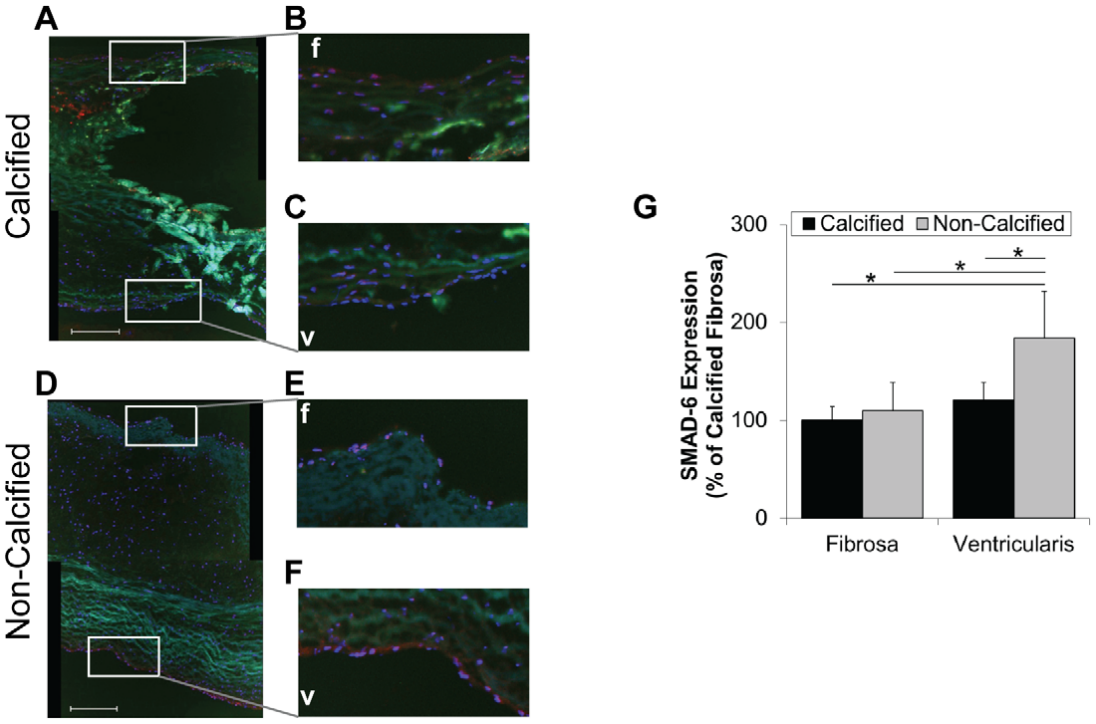
SMAD-6 expression in the fibrosa and ventricularis endothelium. Calcified and non-calcified AV sections were stained for SMAD-6, and a rhodamine-labeled secondary antibody. Shown are representative images. Bar graphs show staining intensities of fibrosa- and ventricularis-endothelium for each antagonist (**G**) (mean+SEM). For SMAD-6, n = 22 calcified and n = 15 non-calcified. *p<0.05, #p<0.06. f: fibrosa, v: ventricularis.

Next, we determined whether the side- and calcification-dependent phosphorylation of SMAD-1/5/8 correlates with expression levels of BMP antagonists (CV-2/BMPER, noggin, DAN, follistatin, chordin, and MGP-1). CV-2/BMPER and noggin expression was significantly lower in the fibrosa endothelium both in calcified and non-calcified AVs (Figure 4 A–G). Furthermore, we found that CV-2/BMPER expression was significantly reduced in the calcified ventricularis endothelium than the non-calcified ventricularis endothelium (Figure 4G), while this disease-dependency was not observed for noggin (Figure 4N). DAN expression was not significantly different in the endothelium; although, a trend of decreased staining was seen between the calcified and non-calcified fibrosa endothelium (Figure 4H–N). At this time, none of the available antibodies that we examined resulted in specific staining patterns for follistatin, chordin, and MGP-1, when compared to isotype IgG controls (data not shown). Lastly, we examined the level of inhibitory SMAD-6. SMAD-6 expression was significantly higher in non-calcified ventricularis endothelium compared to non-calcified fibrosa endothelium and endothelium of calcified valves (Figure 5G).

## Discussion

The main findings of our current work are that 1) phosphorylation of SMAD-1/5/8 is significantly increased in calcified fibrosa endothelium, 2) surprisingly, BMP-2/4/6 expression is higher in the ventricularis endothelium in both calcified and non-calcified AVs, 3) BMP antagonists noggin and CV2/BMPER expression was increased in the ventricularis endothelium in both calcified and non-calcified AVs, and 4) inhibitory SMAD-6 expression was highest in the non-calcified ventricularis endothelium. These results suggest that the side-dependent calcification of human AV is linked to the preferential activation of the BMP-dependent SMAD-1/5/8. This SMAD-1/5/8 activation in the fibrosa endothelium correlates with the decreased levels of the BMP antagonists (noggin and CV-2/BMPER) and inhibitory SMAD-6.

AV calcification and sclerosis primarily occur in the fibrosa, while the ventricularis is relatively unaffected [10], [12]; however, the specific mechanisms underlying this side-dependent AV disease is unclear. Our initial hypothesis was that the side-dependent AV calcification is mediated by preferential activation of BMP signaling pathway in the fibrosa endothelium. To test this hypothesis, we studied SMAD-1/5/8 phosphorylation, the canonical BMP pathway activation marker, by staining frozen sections of AV leaflets obtained from patients undergoing AV replacement (calcified AVs) or heart transplantation (non-calcified AVs) surgeries (Table 1). Our results showed that SMAD-1/5/8 was preferentially activated in the calcified fibrosa endothelium compared to non-calcified fibrosa endothelium (Fig. 2). In contrast, we found no significant differences in phospho-SMAD-2 levels among all groups (Fig. 2), indicating that there was no differential activation of the canonical TGFβ signaling pathway in human AV endothelium. These findings demonstrate a strong correlation between the phospho-SMAD-1/5/8 activation in the endothelium and calcification in the fibrosa side in human AVs.

Previous results have shown clear evidence that disturbed flow stimulates BMP-4 expression in endothelial cells *in vitro*, *ex vivo* and *in vivo*, and that BMP expression leads to endothelial inflammation, aortic wall calcification and AV calcification [21], [24], [25], [27], [38]. In addition, in normal pig AVs, BMP-4 mRNA and protein levels are higher on fibrosa endothelium [15], [20], further demonstrating the close correlation among disturbed flow, BMP-4 expression in the fibrosa endothelium, and side-specific AV calcification.

Therefore, we next tested the hypothesis that the preferential activation of SMAD-1/5/8 on the fibrosa endothelium is mediated by increased BMP expression due to disturbed flow conditions. Contrary to our hypothesis, however, we found that BMP-2,-4 and -6 expression was higher on the ventricularis endothelium compared to fibrosa endothelium of both calcified and non-calcified AV (Figure 3). These results show, unlike in normal healthy porcine AVs, that endothelial BMP expression levels in both calcified and non-calcified human AVs do not correlate with the side-dependent AV calcification and phosphorylation of SMAD-1/5/8. It is interesting, however, that a recent study showed that BMP-4 levels were decreased in the fibrosa endothelium of the hypercholesterolemic pig AVs compared to the normal pig AV [19]. This surprising result in diseased pigs is consistent with our current finding in diseased human AVs.

We next tested the alternative hypothesis that decreased expression of BMP antagonists in the fibrosa endothelium is responsible for the preferential activation of SMAD-1/5/8 and calcification in human AVs. Our results show that noggin and CV-2/BMPER expression levels were higher in the ventricularis endothelium than the fibrosa endothelium in both calcified and non-calcified AVs. These results suggest that abundant levels of BMP antagonists in the ventricularis endothelium, especially in non-calcified human AVs, provide an anti-calcific environment. These results are also consistent with a previous report showing higher chordin mRNA expression on the ventricularis endothelium compared to the fibrosa endothelium in normal porcine AV [20].

In addition to BMP antagonists, BMP pathway can be regulated by inhibitory SMAD-6 and -7 [39]. This led us to test the alternative hypothesis that side-dependent activation of SMAD-1/5/8 and calcification is regulated by inhibitory SMAD-6. We found that SMAD-6 expression levels were highest in the non-calcified ventricularis endothelium, contributing to the anti-calcific environment on the ventricularis-side of the AV (Fig. 5). Consistent with our results, SMAD-6 was shown to be induced by laminar shear stress in vascular endothelial cells [40]. Moreover, SMAD-6 deficiency causes cardiac valve hyperplasia in mice [41], demonstrating its importance in AV biology.

Our results clearly show that the relative lack of the BMP antagonists and inhibitory SMAD-6 in the fibrosa endothelium correlates with the SMAD-1/5/8 phosphorylation in calcified AVs; however, what is not clear is the mechanisms by which SMAD-1/5/8 activation is prevented in non-calcified human AVs compared to the calcified AVs.

There are several potential mechanisms that may explain this finding. First, a recent study showed that patients with AV calcification have significantly lower levels of circulating uncarboxylated MGP (ucMGP) than the healthy cohort [35]. They suggested that the low level of ucMGP was due to the lack of release of MGP into circulation. The deficiency in circulating MGP, in combination with the lack of the BMP antagonists and inhibitory SMAD-6 in the fibrosa endothelium, may promote the side-dependent BMP pathway activation and calcification in human AVs. Second, some BMP antagonists (e.g. follistatin, chordin, MGP-1) that we could not examine due to the lack of specific antibodies for immunostaining studies may also be responsible for our finding. Third, expression of the BMP receptors may also contribute to the observed difference.

It is important to emphasize that the non-calcified AVs used for this study were obtained from recipient hearts following heart transplantations. Therefore, these samples were from heart failure patients, not from a “healthy” subject population, and should not be viewed as non-diseased AVs although they were not calcified. The lack of healthy human aortic valve samples limited our ability to study the factors that may be important in the initiation of this disease. Since our study objective was to test the endothelial expression levels of AV leaflets, we were limited in our approaches to the immunohistochemical staining method since Western blots, using endothelial lysates without significant interstitial contamination, could not be performed. This posed a quantitative and mechanistic limitation in our study.

In summary, we showed that the BMP signaling pathway is preferentially activated in the calcified fibrosa endothelium of human AVs. This side- and disease-dependent activation of BMP pathway correlates with the relative deficiency of the BMP antagonists and inhibitory SMAD-6 in the fibrosa endothelium. These findings suggest that preferential activation of BMP pathways may be controlled by the balance between the BMPs and BMP antagonists and that the BMP inhibitors play a dominant role in side-dependent calcification of human AVs.
